# First Evidence of *Ehrlichia minasensis* Infection in Horses from Brazil

**DOI:** 10.3390/pathogens10030265

**Published:** 2021-02-25

**Authors:** Lívia S. Muraro, Aneliza de O. Souza, Tamyres N. S. Leite, Stefhano L. Cândido, Andréia L. T. Melo, Hugo S. Toma, Mariana B. Carvalho, Valéria Dutra, Luciano Nakazato, Alejandro Cabezas-Cruz, Daniel M. de Aguiar

**Affiliations:** 1 Laboratory of Virology and Rickettsial Infections, Veterinary Hospital, Federal University of Mato Grosso (UFMT), Av. Fernando Correa da Costa 2367, Cuiabá 78090-900, Brazil; liviatcho@hotmail.com; 2 Veterinary Clinical Laboratory, Department of Veterinary Clinics, University of Cuiabá (UNIC), Av. Manoel José de Arruda 3100, Cuiabá 78065-900, Brazil; aneliza-oliveira@hotmail.com (A.d.O.S.); tamyres.leite@hotmail.com (T.N.S.L.); 3 Laboratory of Microbiology and Molecular Biology, Veterinary Hospital of the Faculty of Veterinary Medicine, Federal University of Mato Grosso (UFMT), Av. Fernando Correa da Costa 2367, Cuiabá 78090-900, Brazil; stefanobte@hotmail.com (S.L.C.); valdutra@cpd.ufmt.br (V.D.); lucnaka@mail.com (L.N.); 4 Veterinary of Clinical, Veterinary Medicine College, University of Cuiabá (UNIC), Av. Manoel José de Arruda 3100, Cuiabá 78065-900, Brazil; andreialtm@gmail.com (A.L.T.M.); mama_buenocarvalhos@hotmail.com (M.B.C.); 5 Veterinary Medicine Department, Federal University of Lavras (UFLA), Campus Universitário, Mailbox 3037, Lavras 37200-000, Brazil; hugo.toma@ufla.br; 6 Anses, INRAE, Ecole Nationale Vétérinaire d’Alfort, UMR BIPAR, Laboratoire de Santé Animale, F-94700 Maisons-Alfort, France; alejandro.cabezas@vet-alfort.fr

**Keywords:** horse, ehrlichiosis, ticks, PCR, IFA

## Abstract

The genus *Ehrlichia* includes tick-borne bacterial pathogens affecting humans, domestic and wild mammals. *Ehrlichia minasensis* has been identified in different animal species and geographical locations, suggesting that this is a widely distributed and generalist *Ehrlichia*. In the present study, we evaluated *Ehrlichial* infection in 148 Equidae presented to the Medical Clinic Department of a Veterinary Hospital from a midwestern region of Brazil. Blood samples and ticks collected from the animals were tested by Polymerase Chain Reaction (PCR) for the presence of *Ehrlichia* spp. A multigenic approach including Anaplasmataceae-specific (i.e., 16S rRNA, *groEL*, *gltA*) and *Ehrlichia*-specific (i.e., *dsb* and *trp36*) genes was used for accurate bacteria identification. Sera samples were also collected and evaluated for the detection of anti-*Ehrlichia* antibodies by indirect fluorescent antibody test (IFA). Possible associations between molecular and serological diagnostics and clinical and hematological manifestations were tested using chi-squared or Fisher’s exact tests. Sequence analysis of the *dsb* fragment revealed that three horses (2.03%) were exposed to *E. minasensis*. Sixty-one (41.2%) Equidae (58 equines and three mules), were seropositive for *Ehrlichia* spp., with antibody titers ranging between 40 and 2560. Seropositivity to ehrlichial antigens was statistically associated with tick infestation, rural origin, hypoalbuminemia and hyperproteinemia (*p* ≤ 0.05). The present study reports the first evidence of natural infection by *E. minasensis* in horses from Brazil.

## 1. Introduction

*Ehrlichia* genus is composed of Gram-negative, obligatory intracellular bacteria transmitted by ticks. Several *Ehrlichia* bacteria are important pathogens, causing disease in humans and domestic and wild mammals. This genus belongs to the order Rickettsiales, family Anaplasmataceae, and has six recognized species: *Ehrlichia canis*, *Ehrlichia chaffeensis*, *Ehrlichia ewingii*, *Ehrlichia muris*, *Ehrlichia ruminantium* and *Ehrlichia minasensis* [1,2].

Equine ehrlichiosis has mostly been associated with agents of the genera *Neorickettsia* (i.e., *N. risticii*) and *Anaplasma* (i.e., *A. phagocytophilum*) [1]. In addition to these species, commonly reported to cause Equine ehrlichiosis, infection by bacteria of the genus *Ehrlichia* has also been described in horses in the United States [3,4], and Nicaragua [5]. The genetic characterization of these Ehrlichial agents revealed their close relationship to *E. ruminantium*, which is an important pathogen of ruminants in Africa and the Caribbean [6], transmitted by ticks of the genus *Amblyomma* [7]. However, while Equine ehrlichiosis is present in Brazil [8], to date, there are no reports of *E. ruminantium* in Brazil [9]. *Ehrlichia minasensis* species has been detected in cattle [10], cervids [11] and several tick species within the genera *Rhipicephalus* [2,7,12,13], *Amblyomma* [13], *Hyalomma* [14,15], and *Haemaphysalis* [16]. Of the tick species that could vector this bacterium, *Rhipicephalus microplus* [17], *Amblyomma hebraeum* [13], *Hyalomma marginatum* [13,18,19] and *Haemaphysalis* [18] have been reported to parasitize horses, suggesting that infection by *E. minasensis* could be spread to other hosts, such as equines.

Despite studies on *Ehrlichia* infection in equine being scarce in Brazil, seropositivity to *Ehrlichia* antigens in horses was observed in the States of Paraná [20] and Mato Grosso [21], and the bacterial DNA was also detected in horses of Paraná [8]. Subsequent molecular surveys in Brazil suggest that the Brazilian isolate of *Ehrlichia* detected in horses may potentially belong to the same group as that detected in Nicaragua [5,22].

Given these antecedents and the lack of studies involving Equine ehrlichiosis in Brazil, this study aims to analyze the occurrence of *Ehrlichia* spp. and anti-ehrlichial antibodies in horses from the state of Mato Grosso, as well as to evaluate the possible association between bacterial infection and tick parasitism, clinical manifestations, and hematological changes.

## 2. Materials and Methods

### 2.1. Ethical Statement

The animal use procedures for this study were approved by the Animal Use Ethics Committee of the University of Cuiabá (UNIC), under the protocol number 009/2018.

### 2.2. Tick and Blood Sample Collection and Sera Preparation

One hundred and forty-eight equids (horses n = 142 and mules n = 6) with a history of tick parasitism, that attended the Medical Clinic Service of a Veterinary Hospital, Mato Grosso State, Brazil, between February 2018 and April 2018, were included in the study. The animals were located in ranches of the Cuiabá municipality (15°35′56″ S, 56°5′42″ W) (n = 84 horses), Poxoreu municipality (15°50′27″ S, 54°23′39′ W) (n = 14 horses) and the Poconé municipality (16°7′2″ S 56°57′38″ W) (*n* = 44 horses and six mules)

Blood samples were collected in duplicate tubes: one with EDTA (BD Vacutainer^®^, Franklin Lakes, NJ, USA), one for complete blood count and another for PCR testing (kept at −80 °C). The other tubes, without anticoagulant (BD Vacutainer^®^, Franklin Lakes, NJ, USA), were used for obtaining serum samples. Blood samples without anticoagulant were kept at room temperature (25 °C) until visible clot formation. Subsequently, the samples were centrifuged at 1500× *g* for 5 min, and the obtained sera was used for biochemical and serological evaluation. Samples were kept at −20 °C until further use. 

Ticks, when observed on the animals, were collected and kept in labeled isopropanol tubes until identification according to morphological taxonomic keys for different tick stages [23,24,25].

At the time of collection, an epidemiological questionnaire was applied in order to obtain variables related to the origin (urban or rural) and clinical information such as apathy, fever, mucosal pallor, bleeding, lymphadenemegaly and bloody diarrhea.

### 2.3. Hematological and biochemical Analyses

Hematological analyses were performed using the equipment pocH-100i Vet (Roche, São Paulo, SP, Brazil) and bioplus 200 (Bioplus, Barueri, SP, Brazil). Hematological (i.e., Erythrocytes 5.5–9.5 × 10^6^/µL; hemoglobin 12–18 g/dL; hematocrit 37–55%; white blood cell 6–12 × 10^3^ µL; neutrophils 2.1– 9.0 × 10^3^ µL; eosinophils 0.12–1.44 × 10^3^ µL; lymphocytes 0.9–6.0 × 10^3^ µL; monocytes 0.12–1.2 × 10^3^ µL; platelets 100–350 × 10^3^ µL; total plasma protein (PPT) 6.5–8 g/dL; fibrinogen 100–400 mg/dL) and biochemical (i.e., aspartate aminotransferase (AST) 152-294 IU/L; alkaline phosphatase (ALP) 143–395 IU/L; creatinine 1.2–1.9 mg/dL; urea nitrogen 21.4–51.3 mg/dL; total serum protein (PT) 5.2–7.9 g/dL; albumin 2.6–3.7 g/dL and globulin 2.1–4.0 g/dL) reference parameters adopted by the Clinical Pathology Laboratory of the Veterinary Hospital were used to compared the obtained results. Anemia was considered when the animal showed a decrease in the number of erythrocytes, and/or a decrease in the hemoglobin and/or hematocrit content below the reference value [26].

### 2.4. DNA Extraction and Molecular Detection of Anaplasmataceae Bacteria

The wizard Genomic DNA Purification Kit (Promega, Madison, WI, USA) was used for DNA extraction from whole-blood and tick samples, following the manufacturer’s instructions. Tick DNA was extracted according to Sangioni et al. [27]. In order to monitor the quality of DNA extraction in blood and ticks, samples were selected randomly to perform conventional PCR assays for the mammal endogenous gene glyceraldehyde-3-phosphate dehydrogenase (*gapdh*) [28] and tick mitochondrial 16S rRNA gene [29], respectively.

After DNA extraction in blood and tick samples, nested PCR assays were performed to amplify fragments of Anaplasmataceae 16S rRNA gene (360 base pair) [30], *Ehrlichia* spp. *dsb* (349 bp) [10,31], and Anaplasmataceae *groEL* gene (445 bp) [32]. Positive samples were further evaluated by amplification of Anaplasmataceae *gltA* (650 bp) [33,34] and *Ehrlichia TRP36* genes (800 bp) [10,35]. Primers and annealing temperatures are presented in Table 1.

PCR reactions were prepared with GoTaq Green Master Mix (Promega, Madison, WI, USA) according to the manufacturer’s instructions. The amplification protocol consisted of an initial denaturation step at 95 °C for 5 min, 35 cycles of denaturation (95 °C, 15 s), annealing (52 to 58 °C, 15 s) and extension (72 °C 1 min), followed by an additional extension step at 72 °C for 5 min. Amplification products were mixed with GelRed (Uniscience, São Paulo, SP, Brazil) at 1.5% for visualization, and run on agarose gels in horizontal electrophoresis for 20 min at 120 V. DNA of *Ehrlichia canis* (Cuiabá strain # 1) [35] and ultrapure water were added as positive and negative controls, respectively.

Amplified fragments of the expected size were purified by ReliaPrep DNA Clean-Up and Concentration System^®^ Kit (Promega, Madison, WI, USA) according to the manufacturer’s instructions. Products were then subjected to nucleotide sequencing reaction using the Big Dye Kit (Applied Biosystems, Austin, TX, USA) and 3500 Genetic Analyzer automate sequencer (Applied Biosystems, Austin, TX, USA) according to the manufacturer’s instructions. The sequences obtained were edited in the Geneious software (Biomatters, Auckland, New Zealand). Sequence identity analysis was performed in GenBank using the Basic Local Alignment Search Tool (BLAST).

### 2.5. Serologic Analysis

Sera samples were tested by indirect fluorescent antibody test (IFA) using *E. canis* Cuiabá # 1 strain antigens. The bacterium was cultured in DH82 cells as previously described [21]. Dilutions of the sera samples at 1:40 to 1:5120 were prepared in PBS (pH 7.2) and applied to the IFA slides containing the antigen previously fixed using Acetone. Negative and positive control sera were included in each slide. Rabbit anti-equine IgG conjugated with FITIC (Sigma Diagnostics, St. Louis, Mo, USA) was added at dilution 1:800. Glycerin (pH 8.5) was added to each slide to coverslip. Seroreactions were visualized under an epifluorescence microscope (Scope.A1 Zeiss) using a 40× objective.

### 2.6. Statistical Analysis

For statistical analysis, the R software (R Development Core Team, 2010) was used. Statistical evaluation was based on the association between clinical findings, laboratory tests, and any positive results for PCR or IFA, as well as for both of them. Therefore, we considered the dichotomous variable named “result”, which assumed that the value of 1 in horses was characterized as positive in IFA and PCR, and the value of 0 as negative. 

To calculate the association measure used for qualitative variables, the Chi-square test (χ2) and Fisher’s exact test were used when necessary, with a 95% confidence interval. For the numerical variables, the normal distribution of the data was verified by the Kolmogovov–Smirnov test, and the Mann–Whitney test was applied due to the non-normal distribution. Differences were considered significant at *p* ≤ 0.05. 

## 3. Results

### 3.1. Health Status of the Equidae Used in the Study

From the 148 evaluated equidaes, 70 (47.4%) were males and 78 (52.7%) females, 142 (95.9%) were horses and six (4.1%) were mules. The clinical questionnaire revealed clinical manifestations such as apathy (1.35%), anemia (2.03%) and fever (0.7%) in some animals (Table 2). No animal presented hemorrhage, diarrhea or lymphadenomegaly.

### 3.2. Tick Infestation

Ticks were observed and removed from 89 animals (60.14%), of which 88 were horses and one a mule (Table 2). Seventy-eight (8.7%) ticks were identified as *Amblyomma sculptum* (37 males and 41 females), 821 (91.1%) as *Dermacentor nitens* (83 males, 460 females and 278 nymphs) and 2 as *Rhipicephalus microplus* (both males) (Table 3).

### 3.3. Hematological Parameters of Blood Samples

The hematological changes observed were anemia in 101 (68.2%) animals, leukocytosis in 39 (26.3%), neutrophilia in 11 (7.4%), lymphocytosis in 26 (17.6%), plasma hyperproteinemia in 47 (31.7%) and hyperfibrinogenemia in 59 (40.1%). Fourteen (9.4%) animals had azotemia, 77 (52.0%) had elevated AST and only 9 (6.1%) had increased ALP; hypoalbuminemia was seen in 36 (24.3%) and hyperglobulinemia in 72 (48.6%).

### 3.4. Molecular Detection of Anaplasmataceae Bacteria

The *gapdh* and tick mitochondrial 16S rRNA gene were consistently amplified from DNA horse and tick samples. Amplicons for *dsb* gene were detected in blood samples of three horses (2.03%) and were negative for the 16S rRNA gene. Sequence analysis of the *dsb* fragments revealed a sequence 99–100% identical to *E. minasensis* sequences (GenBank accession numbers: MH500007, KX258450, KT970783, KT314243, KF621012). The *dsb* sequences generated in this study were deposited into GenBank and assigned the nucleotide accession numbers of MT232423, MT232424, MT232425. These three samples were negative for the *groEL*, *trp36* and *gltA* genes. All ticks were also negative in the PCR assays.

### 3.5. Detection of Anti-Ehrlichia IgG

Anti-*Ehrlichia* spp. IgG was detected in 61 (41.2%, total 148) samples (58 equines and three mules), and the antibody titers registered were 40 (22.95%, 14, total 61), 80 (45.9%, 28, total 61), 160 (11.5%, 7, total 61), 320 (13.1%, 8, total 61), 640 (3.27%, 2, total 61), 1280 (1.64%, 2, total 61) and 2560 (1.64%, 2, total 61). Positive samples were observed in 17 (20.2%) small ranches from Cuiabá, and one (100%) farm from Poconé and Poxoreu.

### 3.6. Association between Exposure to Ehrlichia Infection and Clinical and Hematological Manifestations

The results of χ2 test showed that three (2.03%) PCR-positive animals were associated with the rural environment (*p* = 0.04) and hyperproteinemia (*p* = 0.03). The tendency of association was observed between PCR and hyperfibrinogemia (*p* = 0.06). Associations were found between IFA-positive animals and rural origin (*p* = 0.0001), anemia (*p* = 0.01), hyperproteinemia (*p* = 0.00001), hypoalbuminemia (*p* = 0.02), normal albuminemia (*p* = 0.05), and the presence of ticks (*p* = 0.03). Table 4, Table 5 and Table 6 show the results of statistical analyses.

## 4. Discussion

This paper reports, for the first time, the molecular detection of *E. minasensis* in horse blood in Brazil. *Ehrlichia minasensis* was first described in cattle from Canada [36], and later in *R. microplus* ticks and cattle from Brazil [7,10,36,37]. Additionally, *E. minasensis* has been the subject of studies on morphological and genetic characterization [38] and their distribution has been shown in recent reports in Corsica, France [14], Pakistan [15], Ethiopia [39], South Africa [13], Israel [40] and China [16].

Our result reinforces the hypothesis that this ehrlichial species is not restricted to cattle. Indeed, previous reports detected *E. minasensis* DNA in deer samples in Canada [11]. Horses can be infected while grazing on infested *R. microplus* pasture or when raised close to bovines [17]. Although no positive ticks for *E. minasensis* were detected, a previous study showed that populations of *A. sculptum* ticks exposed to naturally infected cattle, were negative by PCR. In contrast, *R. microplus* ticks were positive under the same conditions [12].

In this sense, even under a low frequency of *R. microplus* among Equidae in the present study, the transmission of *E. minasensis* by this tick species must be considered. In addition, the three positive PCR samples were from animals from a single farm, which suggests a source of infection with epidemiological relevance for horses in this site. 

In this context, approximately 70% of seropositive equids were infested with at least one tick species, and these animals had 2.1 chances to obtain anti-*Ehrlichia* spp. antibodies (Table 4). Our result corroborates results in the USA [4], which found greater positivity in horses parasitized by ticks.

Seropositives Equidae were found in 41.2% of samples and titers of anti-*Ehrlichia* spp. antibodies ranging from 40 to 2560. The presence of serum immunoglobulins against ehrlichia was previous reported in horses from the Pantanal wetlands [21], but with lower frequency and antibody titers. Serological surveys of ehrlichial infection in equids in Brazil are scarce; another study reported the presence of anti-*Ehrlichia* spp. antibodies in horses from a rural settlement in the Parana state, Southern Brazil, with similar IFA antibody frequencies to the present study [20].

In line with the risk observed with tick’s parasitism, animals from a rural environment had a 7.9 chance of being seropositive compared to animals living in urban environments. This association reinforces the possibility of transmission by *R. microplus* ticks, since the presence of cattle in urban areas is usually insignificant when compared to their presence in the rural environment.

Hematological variables, such as anemia, hyperproteinemia and hypoalbuminemia, were associated with seropositivity for *Ehrlichia* spp. This result agrees with the clinical and laboratory characteristics of ehrlichiosis, whether in dogs, cattle or humans, that were usually found to have a significant hyperproteinemia [10,41,42]. In this study, positive horses had 5.2 chances of having high plasma protein compared to negative horses (*p* < 0.05). Hyperproteinemia with hyperglobulinemia is a common abnormality found in dogs with canine monocytic ehrlichiosis, due to continuous prolonged antigen stimulation [42]. In this study, hyperglobulinemia (33/61) was observed in positive equid; however, statistical significancy was not detected. 

The anemia variable can be explained by the inflammatory response when serum iron is unavailable, which compromises erythropoiesis. Significant decreases in iron levels were identified in horses with local and systemic inflammatory disease [43]. Although the albumin variable showed a statistical result (*p* < 0.05), the highest observed frequency was between normal albumin values, followed by hypoalbuminemia. Thus, in the present study, this result does not reflect any laboratory parameter for equine ehrlichiosis. However, hypoalbuminemia can be observed in several situations, such as chronic liver damage, a deficit of intestinal protein absorption due to helminths, and kidney disease [44].

Increased serum fibrinogen levels were observed in both PCR/IFA-positive animals, because the highest fibrinogen levels were more frequent among the positive horses. However, these increases were not statistically significant (*p* = 0.06/*p* = 0.11), but the results suggest the tendency of an increase in serum fibrinogen, observed in positive animals. Elevated fibrinogen, occurring in horses, is relevant in acute phases of infections [45,46], and this result denotes an inflammatory response related to the positivity to diagnosis ehrlichiosis seen in both assays in the present study. Additionally, hyperfibrinogenemia can be justified by animals presenting plasma hyperproteinemia [26].

## 5. Conclusions

The present study reports the first evidence of natural infection by *E. minasensis* in horses in Brazil. Additionally, seropositivity against *Ehrlichia* antigen was associated with parasitism by ticks, rural origin and anemia. Hyperproteinemia and hyperfibrinogenemia were detected in most positive animals, reflecting inflammatory response. In the absence of a statistically significant association between the positivity of animals and clinical signs, further studies of *Ehrlichia* spp. infection in horses should be performed to better understand their relevance in the natural history of this ehrlichiosis in Brazil.

## Figures and Tables

**Table 1:** Primers used in the study.

Genes	Primer Identification	Sequence (5′–3′)	Annealing Temperature	Bp	References
*16S* rRNA *	Ge2F2	GTTAGTGGCAGACGGGTGAGT	58.0 °C	360	[30]
	He3	TATAGGTACCGTCATTATCTTCCCTAT			
dsb *	DSB330 F ^1^	GATGATGTTTGAAGATATSAAACAAAT	50.0 °C	349	[31]
	DSB720 R ^1,2^	CTATTTTTACTTCTTAAAGTTGATAWATC			
	DSB380F ^2^	ATTTTTAGRGATTTCCAATACTTTGG	50.4 °C		
groEL *	GRO 607 F ^1^	GAAGATGCWGTWGGWTGTACKGC	57.0 °C	445	[32]
	GRO 1294 R ^1^	AGMGCTTCWCCTTCWACRTCYTC			
	GRO 677 F ^2^	ATTACTCAGAGTGCTTCTCARTG	57.0 °C		
	GRO 1121 R ^2^	TGCATACCRTCAGTYTTTTCAAC			
TRP36 **	TRP36F2 ^1^	TTTAAAACAAAATTAACACACTA	52.0 °C	800	[35]
	TRP36R1 ^1,2^	AAGATTAACTTAATACTCAATATTACT			
	TRP36DF ^2^	CACACTAAAATGTATAATAAAGC	57.0 °C		[10]
*glt*A **	F4b ^1^	CCGGGTTTTATGTCTACTGC	55.0 °C	650	[34]
	R1B ^1^	CGATGACCAAAACCCAT			[33]
	EHRCS136F ^2^	TTYATGTCYACTGCTGCKTG	55.0 °C		
	EHR778R ^2^	GCNCCMCCATGMGCTGG			

^1^ First reaction, ^2^ Second reaction, * Horse and tick DNA, ** primers used in positive samples.

**Table 2:** Epidemiological and clinical data from equids evaluated for *Ehrlichia* infection.

Parameters	Number of Animals	%
Origin		
Rural	65	43.9
Urban	83	56.1
Clinical manifestations		
Apathy	2	1.35
Fever	1	0.7
Pale mucous membranes	3	2.03
Bleeding	0	0.0
Lymphadenomegaly	0	0.0
Bloody diarrhea	0	0.0
Tick infestation	89	60.14

**Table 3:** Ticks collected from equids evaluated for *Ehrlichia* infection.

Tick Species	Number of Ticks				
	Male	Female	Nymph	Total	%
*Amblyomma sculptum*	37	41	0	78	8.7
*Dermacentor nitens*	83	460	278	821	91.1
*Rhipicephalus microplus*	2	0	0	2	0.2

**Table 4:** Associations between indirect fluorescent antibody (IFA)/polymerase chain reaction (PCR)-positivity to *Ehrlichia* and the clinical manifestations.

Variables	Equidae			χ2		
	Tested	Positive *	%	Odds Ratio	IC 95%	*p*
Species						
Mule	6	3	50	1.4	0.18–11.16	0.69
Horse	142	58	40.8			
Sex						
Female	78	32	41.0	1.01	0.5–2.0	0.9
Male	70	29	41.4			
Origin **						
Urban	83	17	20.5		3.6–18.3	0.0001/0.04
Rural	65	44	67.7	7.9		
Ticks infestation	89	43	48.3	2.1	1.0–4.5	0.03
Apathy	2	1	50.0	1.4	0.01–113.7	1
Fever	1	0	0	0	0.0–55.57	1
Pale mucous membranes	3	2	66.7	2.9	0.14–173.7	0.5
Bleeding	0	0	0	-	-	-
Lymphadenomegaly	0	0	0	-	-	-
Diarrhea with blood	0	0	0	-	-	-
Anemia	101	35	34.65	---	---	0.01

* Positive for PCR and IFA; ** PCR *p* = 0.04/IFA *p* = 0.0001.

**Table 5:** Associations between IFA/PCR-positivity to *Ehrlichia* and the haematological parameters.

Parameter	Equidae				
	*N*	PCR (%)	*p*	IFA (%)	*p*
**Hematocrit (%)** <37 37–55 >55	97 50 1	3 (2.03) 0 (0) 0 (0)	0.55 0.18 1.0	35 (36.1) 26 (52.0) 0 (0)	0.08 0.05 1.0
**Erythrocytes (× 10^6^/µL)** <5.5 5.5–9.5 >9.5	14 113 21	0 (0) 3 (2.6) 0 (0)	1.0 1.0 1.0	6 (42.8) 49 (43.4) 6 (28.6)	0.89 0.34 0.20
**Hemoglobin (g/dL)** <12 12–18 >18	95 52 1	2 (2.1) 1 (1.9) 0 (0)	1.0 1.0 1.0	34 (35.8) 27 (51.9) 0 (78.6)	0.07 0.05 1.0
**Leukocyte count (× 10^3^ µL)** <6.0 6.0–12.0 >12.0	2 106 40	0 (0) 1 (0.9) 2 (5.0)	1.0 0.19 0.16	1 (50.0) 43 (40.6) 17 (42.5)	1.0 0.79 0.72
**Neutrophils (× 10^3^ µL)** <2.1 2.1–9.0 >9.0	0 137 11	0 (0) 3 (2.2) 0 (0)	1.0 1.0	0 (0) 58 (42.3) 3 (27.3)	0.11 0.11
**Lymphocytes (× 10^3^ µL)** <0.9 0.9–6.0 >6.0	2 120 26	0 (0) 3 (2.5) 0 (0)	1.0 1.0 1.0	2 (100.0) 49 (40.8) 10 (38.5)	0.16 0.70 0.89
**Monocytes (× 10^3^ µL)** < 0.12 0.12–1.2 >1.2	97 50 1	2 (2.1) 1 (2.0) 0 (0)	1.0 1.0 1.0	41 (42.3) 20 (40.0) 0 (0)	0.72 0.85 1.0
**Eosinophils (× 10^3^ µL)** <0.12 0.12–1.44 >1.44	32 115 1	0 (0) 3 (23.5) 0 (0)	1.0 1.0 1.0	12 (37.5) 49 (70.6) 0 (0)	0.62 0.52 1.0
**Platelets (× 10^3^ µL)** <100 100–350 > 350	11 132 5	0 (0) 3 (2.3) 0 (0)	1.0 1.0 1.0	4 (36.4) 55 (41.7) 2 (4)	1.0 0.74 1.0
PPT * <6.5 6.5–8.0 >8	6 95 47	0 (0) 0 (0) 3 (6.4)	1.0 0.04 0.03	1 (16.7) 28 (29.5) 32 (68.1)	0.40 0.0001 0.00001
Fibrinogen ** <100 100–400 >400	0 88 59	0 (0) 0 (0) 3 (5.1)	0.08 0.06	0 (0) 31 (35.2) 29 (49.2)	0.13 0.11

* PPT–Total Plasma Protein; ** It was not performed on a horse.

**Table 6:** Associations between IFA/PCR-positivity to *Ehrlichia* and the biochemical parameters.

Biochemicals	Equidae				
	*N*	PCR (%)	*P*	IFA (%)	*P*
AST *					
<152 152–294 >294	6 65 77	0 (0) 0 (0) 3 (3.9)	1.0 0.41 0.24	3 (50.0) 27 (54.0) 31 (40.3)	1.0 0.61 0.80
ALP ** <143 143–395 >395	19 120 9	0 (0) 3 (2.5) 0 (0)	1.0 1.0 1.0	7 (36.8) 52 (43.3) 2 (22.2)	0.67 0.27 0.30
**Urea nitrogen** <21.4 21.4-51.3 >51.3	10 130 8	0 (0) 3 (2.3) 0 (0)	1.0 1.0 1.0	2 (20.0) 55 (42.3) 4 (50.0)	0.19 0.46 0.71
**Creatinine** <1.2 1.2–1.9 >1.9	29 105 14	1 (3.4) 2 (1.9) 0 (0)	0.41 1.0 1.0	12 (41.4) 45 (42.9) 4 (28.6)	0.96 0.52 0.31
PT *** <5.2 5.2–7.9 >7.9	21 91 36	0 (0) 2 (2.3) 1 (2.8)	1.0 1.0 0.56	6 (28.6) 37 (40.7) 18 (50.0)	0.20 0.97 0.20
**Albumin** <2.6 2.6–3.7 >3.7	36 101 11	1 (2.8) 2 (2.0) 0 (0)	0.56 1.0 1.0	9 (25.0) 47 (46.5) 5 (45.5)	0.02 0.05 0.76
**Globulin**					
<2.6 2.6–4.0 >4.0	26 50 72	0 (0) 2 (4.0) 1 (1.4)	1.0 0.23 1.0	6 (23.1) 22 (44.0) 33 (45.8)	0.03 0.52 0.26

* AST–Aspartate aminotransferase; ** ALP–Alkaline phosphatase; *** PT–Total serum protein.

## Data Availability

Not applicable.
